# Miscellaneous

**Published:** 1858-03

**Authors:** 


### CHEOPLASTY.

Messrs. Editors—will you give an old and retired dentist space in your valuable periodical to say a few words about Cheoplasty. When I first noticed this method for inserting teeth, I supposed that the shrinkage of the metal would prevent a good fit, and that to get strength it would be clumsy. I also supposed that the metal would corrode in the mouth. My short experience in the use of an upper denture for myself has convinced me of my error.

Dr. Blandy about two weeks ago made me an upper set which for accuracy of fit, use and comfort in the mouth is far superior to any I have ever had. This was accomplished the first casting, and is not at all clumsy, nor has it tarnished or give any unpleasant taste in the mouth. In fine it is usefull, sweet and cleanly.

I think that Dr. Blandy has been very fortunate in geting a combination of metals which appear to meet all the demands of mechanical dentistry and can not fail to be generally introduced. Yours truly, &c.,

Joseph Taylor.

---

### "VALERIANATE OF AMMONIA."

Seeing in the "Register" among the extracts of the last number a notice of the above which is not very definite, it may not be out of place here to give an extract from "Braithwatie's Retrospect." "In a subsequent communication, Dr. Decalt has stated that the Valerianate of Ammonia which he employs, is a brown liquid, not very limpid, of a disagreeable taste, and smelling strongly of the peculiar oder of Valerian." This is the Tinctura Valerianæ Composta of the United States Dispensatory. Having used it in a number of severe cases of neuralgia, I have realized from it my fullest anticipations. I also used it in cases of "sick head-ache." One case in which the patient was laid up every week or two, which it seems other medicines could not arrest short of one or two days confinement and severe suffering. The patient now takes about two tea-spoonsfull about three or four hours apart, when she finds the dullness of the eyes coming on, and has not been laid up since.

Bellfonte, Pa. J. D. WINGATE.

### MOULDING SAND—TO IMPROVE IT.

One day after moulding a difficult model, the idea of mixing asbestos with the sand, to improve its adhesibility occurred to me. Having, after soldering, thrown my asbestos investments into an empty vessel for some time, I now took them out and pulverized them in a morter, and sifted the plaster and fine asbestes into my sand tray and find that the sand is much improved. The metal casts come out much smoother, as it does not burn. The asbestos remaining in the seive is improved and ready for use again. Would not the grinding up of old plaster models make a good substitute for sand? J. D. WINGATE.

---

### TREATMENT OF EXPOSED PULP.

BY W. R. LILLY.

In looking over the reports of the proceedings of the dental convention of Northern Ohio, I noticed a resolution in regard to the treatment of exposed dental pulp—which brought to my mind a case that I had a few months since, and it occured to me that perhaps it might be of some little importance in a table of statistics, such as you spoke of in the last Register, and as you requested members of the profession who were not at the convention, to adopt the suggestion of the resolution, I will give you the facts in this case, and you can make what disposition you like of them.

Miss G., age 14 years, nervous temperment, health good, called to have some teeth filled, October 8th, 1857, in preparing a cavity on the anterior approximal surface of the left upper first molar, I exposed and wounded the pulp which bled some at the time, but not much, after the hemorrhage ceased I applied some chloride of zinc for a few minutes, after which I finished forming the cavity. I then applied a pledget of cotton satnrated with a coucentrated solution of tanic acid and closed the cavity with wax and let it remain whilst I prepared and filled another tooth, I then made a small cap of some six or eight thicknes es, of No. 6 foil, after warming the cap I touched it with a bit of bees-wax, to give it a thin coating on one side, I then removed the tannin and touched the exposed nerve with a little creosote. Then after drying the cavity I applied my cap with the waxed side immediately over the exposed nerve, and over this I laid some asbestos and then filled the cavity with gold The tooth gave some little trouble for a week or so, but at present is doing well, I examined the tooth a few days ago and found it alive and apparently in a good condition.

This as a single case is of no importance, but a number together, both successful and unsuccessful, would be interesting.

I have had another case under treatment for the last two weeks, it has given me more trouble than it is worth already, and the indications at present, are favorable for a failure, the patient in this case is some fifty years of age, and the treatment similar to the first, so that it would seem that the chance of success is in favor of young persons.

---

### WESTERN DENTAL SOCIETY.

The undersigned, a committee, appointed in May last, to prepare and announce the subjects, and order of discussion at the next meeting, to commence on the 22d July next, at Quincy, Illinois, respectfully submit that the discussions come under the head of "miscellaneous business," and in the following order:

1. Difficult Dentition.
2. Cause and treatment of the irregularity of the teeth.
3. Best method of treating diseased dentine when the pulp is exposed, or destroyed by natural causes.
4. Filling Teeth.
5. Best manner of inserting partial sets with clasps.
6. Relative merits and demerits of the various modes of constructing artificial dentures.

We respectfully urge upon members and visitors, having reports of cases, casts, cuts, diagrams, or specimens of operations, or work, together with new instruments, tools, fixtures or designs, intended to promote the interests, or facilitate the practice of our profession, to present them, for inspection, before the society.

Come gentlemen, let each one bring something for the good of the whole.

H. E. PEEBLES,

G. H. PERINE,

A. BLAKE, *Committee.*

### DR. BLAKESLEY'S METHOD OF FILLING TEETH.

"It may be laid down as a maxim, in filling teeth, that the cavity, in the first place, should be perfectly freed from all decay, and that it should be of such a shape as will prevent the material used for filling from escaping. In order to secure this result, it in necessry that the interior of the cavity should be of larger dimensions than the orifice. The Crystal Gold Foil can be welded, in such a cavity, into so solid a mass, that it is impossible to separate or displace it except by violent means. It will not crumble and fall away in particles, *if properly inserted*, which all other gold foils, (according to my experience,) are liable to do. In order to secure this solidity, I deem the use of comparatively small instruments essential, as by their use the newly introduced portions of foil may be more thoroughly incorporated with that already in the cavity.

" In describing my mode of preparing the tooth, I shall, in order to adapt the description to the various positions of cavities, use the term bottom to indicate that surface of the cavity which is opposite the opening, whether that opening be upward, downward or lateral. After having thoroughly cleansed the cavity, I proceed to give it the shape as before stated; which I accomplished with small English broaches of suitable length, by slightly drilling or perforating the anterior and posterior angles of the upper part of the cavity, and also in the lower angle. I then connect them by drilling along the circumference between the enamel and the bony structure of the tooth, and also by means of a diamond pointed excavator, cut ting in the same manner; thus forming an entire groove around the cavity. In cases where the walls of the cavity are frail, I rely almost entirely on these grooves for the retention of my filling, making them of considerable depth, so that when the filling is introduced, it is literally dove-tailed; the portions of gold occupying these groves form projections from the main mass, with which they are thoroughly incorporated, thus rendering escape impossible.

" To prepare my gold for filling, I take a sheet of Foil, (say of No. 4), and with my hands perfectly dry, lightly roll it into the shape of a rope, I take halves, thirds and quarters, and treat in the same manner. These I cut into square lengths, and thus have a series of pellets, from moderate size down to that of a small pin head.

" I commonly require about three sizes of filling instruments, turned at different angles, to suit any and every case that may be presented; the points of those in most constant use, will just enter respectfully Nos. 22, 24 and 26 of the wire guage. The end of the smallest is cut with a screw-head file, into three well defined, rather delicate points, not too deep, and that of the larger into four points, observing the same precaution concerning the depth, or else the instrument will not disengage itself without tearing away part of the gold, while attempting to introduce it, thus choking the instrument and rendering it useless until freed of its burden.

"I commence to fill by inserting as large a pellet of gold as will occupy the upper part of the cavity; and continue adding pellets from side to side, until one-half of the bottom is covered; I then apply moderate pressure by direct and laterial force till the whole is tolerably condensed. I then endeavor to free it from the wall of the cavity at its edge. If I find I can do this, into the artificial cavity thus formed, I introduce other small pellets until it is completely filled—hard and solid. I repeat the operation on the opposite edge, and then firmly condense this portion of the filling, which now lies immovable and in a fit condition to be welded upon. I then proceed to fill the other half of the cavity, in the same manner, commencing from the lower part of the cavity, taking great precaution to have this portion well filled, as it is one of the most important points; and make firm the line of junction of the two halves, by forcing in small pellets wherever I can make a depression. I then weld on particles of gold until the cavity is filled up flush with the surface of the tooth, and when it is desireable, add on in such direction and quantity as to restore the original outline. In some cases of longitudinally fractured incisors, I have thus built on an addition of one-third or one-half of the original width of the tooth. My patients often express great delight at this perfect restoration of the shape of their teeth.

"The above is more particularly a description of cavities in a lateral position of the incisors; the same principle, however, holds good in all other cases, simply varying their position. When I have a very large cavity, for instance in a molar tooth, I sometimes commence by inserting whole, half or quarter sheets of foil, lightly formed into pellets of an oval or globular shape. In such cases, I frequently use a wedge-shaped instrument, serrated at the point; finishing off as in ordinary cases with smaller pellets. I conclude the operation as usual, by polishing, "giving a surface," which this Foil receives to an eminent degree.

"Some operators have said that the crystal gold foil hardens too quickly. I experience no trouble from this source, as I avoid applying sufficient pressure to render it unyielding until I am ready to condense it.

"It is important to avoid, when this gold is used, a rounded shape of the cavity, as such a shape has a tendency to allow a pellet to escape from under the instrument, when you attempt to apply another one to it, while an angular shape serves to prevent this.

"To sum up, I would say, avoid the use of too large instruments, avoid introducing the gold in too large masses, and avoid applying pressure till the gold is where you want it to condense and harden.

" I consider the property of welding, which A. J. WATTS & Co.'s crystal gold and crystal gold foil possesses, as adding greatly to their value to the dental operator. It enables him to restore the lost shape of a tooth, and by taking care to secure proper points of support, to fill cavities and save teeth incapable of being reached by other foils."

We will add that neither Dr. BLAKESLEY, nor any other Dentist with whom we have conversed, finds it necessary to anneal our foil just previous to use, as it is already in a fit state to weld when it is received from the manufacturers.

We can supply such instruments as Dr. BLAKESLEY uses, to those of the profession who desire them. A. J. WATTS & CO.

UTICA, November 1, 1857.

---

### BENDING PLATES.

If you lay a piece of thin and *stout* cloth, three or four inches square between the plate and a well formed female cast, before "striking up," you may after striking, lift the plate out with ease.

BENTONVILLE, Ind. MERCHANT KELLY.

---

*At a meeting of the Graduating Class of the Baltimore College of Dental Surgery, held January* 22, 1858, *the following Preamble vnd Rosolutions were offered and unanimously adopted.*

WHEREAS, we, the Graduating Class of the Baltimore College of Dental Surgery, of the Session of 1857–'58, deeming it desirable to have placed within the College Edifice some lasting tribute to the memory of our late Prof. of Anatomy, Dr. WASHINGTON R. HANDY; and feeling sure that all who have owed some portion of their Dental Education to his valuable instructions, would be gratified by having the opportunity of contributing to honor his memory: it is, therefore,

*Resolved*, That we, as a body, take proper measures to collect from all previous graduates of this College now living, as well as from ourselves, a sufficient sum to procure a "half-length" Portrait of Prof. Handy, which shall be presented to the Baltimore College of Dental Surgery; and also,

*Resolved*, That we appoint Dr. C. B. Harris to correspond with the graduates of the College to collect funds for the purpose above specified, and also for the purpose of procuring and sending to each contributor a Photograph Copy of the Portrait.

*Resolved*, That no contributions be received exceeding ten dollars.

*Resolved*, That Dr. C. A. Harris, President of the College, be requested to engage the Artist, and superintend the painting of the picture.

*Resolved*, That our proceedings in this matter be published in the Dental Journals.

James G. Russell, M. D., Me.; J. Smith Dodge, Jr., M.D., N. Y.; Henry Clarke, Md.; Juan N. Boza, Cuba; Alphonse L. Cartier, M. D., Switz.; Samuel H. Beard, Ala.; Thomas O. Hills, D. C.; Chas. W. Cadden, M. D., Md.; Vines E. Turner, N. C.; Luis M. Diaz, Cuba; Fernando Z. Bazen, Cuba; Geo. P. Woodbury, Mass; Thornton W. Tomlinson, Va.; Cornelius S. Hurlburt, Mass.; Carver W. Brown, Va.; Edward D. Hamner, Va.; Nimrod W. Long, Ala.; Armand F. Bignon, M. D., Ga.; Middleton T. Henkel, M. D. S. C.; Nathaniel H. Gibbes, M. D., S. C.

C. B. HARRIS, *Correspondent.*

